# Impairment of Mitochondrial Respiration in Metabolic Diseases: An Overview

**DOI:** 10.3390/ijms23168852

**Published:** 2022-08-09

**Authors:** Vlad Florian Avram, Adrian Petru Merce, Iasmina Maria Hâncu, Alina Doruța Bătrân, Gabrielle Kennedy, Mariana Georgeta Rosca, Danina Mirela Muntean

**Affiliations:** 1 Department VII Internal Medicine—Diabetes, Nutrition and Metabolic Diseases, “Victor Babeș” University of Medicine and Pharmacy, Eftimie Murgu Sq. No. 2, 300041 Timișoara, Romania; 2 Center for Molecular Research in Nephrology and Vascular Disease, “Victor Babeș” University of Medicine and Pharmacy, Eftimie Murgu Sq. No. 2, 300041 Timișoara, Romania; 3 Doctoral School Medicine—Pharmacy, “Victor Babeș” University of Medicine and Pharmacy, Eftimie Murgu Sq. No. 2, 300041 Timișoara, Romania; 4 Center for Translational Research and Systems Medicine, “Victor Babeș” University of Medicine and Pharmacy, Eftimie Murgu Sq. No. 2, 300041 Timișoara, Romania; 5 Department of Foundational Sciences, Central Michigan University College of Medicine, Mount Pleasant, MI 48858, USA; 6 Department III Functional Sciences—Pathophysiology, “Victor Babeș” University of Medicine and Pharmacy, Eftimie Murgu Sq. No. 2, 300041 Timișoara, Romania

**Keywords:** obesity, diabetes mellitus, insulin resistance, mitochondrial respiration, high-resolution respirometry

## Abstract

Mitochondrial dysfunction has emerged as a central pathomechanism in the setting of obesity and diabetes mellitus, linking these intertwined pathologies that share insulin resistance as a common denominator. High-resolution respirometry (HRR) is a state-of-the-art research method currently used to study mitochondrial respiration and its impairment in health and disease. Tissue samples, cells or isolated mitochondria are exposed to various substrate-uncoupler-inhibitor-titration protocols, which allows the measurement and calculation of several parameters of mitochondrial respiration. In this review, we discuss the alterations of mitochondrial bioenergetics in the main dysfunctional organs that contribute to the development of the obese and diabetic phenotypes in both animal models and human subjects. Herein we review data regarding the impairment of oxidative phosphorylation as integrated mitochondrial function assessed by means of HRR. We acknowledge the critical role of this method in determining the alterations in oxidative phosphorylation occurring in the early stages of metabolic pathologies. We conclude that there is a mutual two-way relationship between mitochondrial dysfunction and insulin insensitivity that characterizes these diseases.

## 1. Introduction

Mitochondria are central organelles that provide energy via the process of oxidative phosphorylation (OXPHOS), which produces adenosine triphosphate (ATP), the main cellular energy currency [1]. This is a complex, highly-coordinated process that requires the oxidation of NADH or FADH_2_ generated via glycolysis, Krebs cycle or β-oxidation of fatty acids, with the metabolic flux being driven by the ATP demand. Mitochondrial function or mitochondrial respiratory capacity refers to the capability of mitochondria to generate ATP in order to match the cellular demand. Conversely, mitochondrial dysfunction in bioenergetics refers stricto sensu to the inability of mitochondria to appropriately generate ATP in response to energy demands; however, in a wider understanding, several other functions such as impaired redox and calcium homeostasis, intermediary metabolism and cell death regulation are also included under the umbrella of mitochondrial dysfunction [2]. 

Research from the past decades has shown that impaired bioenergetics is implicated in the pathophysiology of a large spectrum of diseases [3,4,5,6], and pharmacological targeting of the energy metabolism is a promising therapeutic approach in these conditions, including obesity [7,8,9,10,11,12]. 

Metabolic diseases are characterized by the impairment of carbohydrate and lipid metabolism and numerous comorbidities that ultimately lead to premature death. A plethora of studies support the hypothesis that mitochondrial dysfunction is a key pathomechanism that links obesity and type 2 diabetes (T2D), both conditions being characterized by insulin resistance [13,14,15,16]. The decrease in OXPHOS is most frequently associated with an increase in mitochondrial reactive oxygen species (ROS) generation, which has been linked to the onset and progression of biological changes accompanying the cardiometabolic pathologies.

Obesity has been declared by the World Health Organization (WHO) as an ongoing epidemic of the 21st century, a disease “with no borders” whose prevalence has nearly tripled since the 1970s, thus posing a huge social and health system burden worldwide. Despite tremendous research efforts, ‘globesity’ (a term coined by WHO two decades ago) continues to spread around the world, and is the major risk factor for the most frequent non-communicable diseases (NCD), namely T2D and cardiovascular diseases. While there is a clear causal link between obesity and diabetes, it is still unclear why only some obese persons develop diabetes. 

Mitochondrial respiratory dysfunction is a common denominator in many NCD, and supports both the decrease in energy utilization and insulin insensitivity, the signature of T2D [15,17]. Current estimates place around 537 million people as suffering from diabetes worldwide, mostly T2D, a worrisome fact that is strongly entwined with the global rise in obesity prevalence [18]. Diabetes mellitus is a chronic condition in which decreased insulin activity, manifested as either tissue insulin insensitivity or insulin amount, causes reduced glucose disposal [18,19]. In long term, chronic hyperglycemia, metabolic abnormalities (gluco- and lipo-toxicity) and oxidative stress eventually lead to progressive β-cell loss and failure with subsequent decreased insulin secretion, which further impairs the glycemic control [20,21]. 

Obesity and diabetes are components of metabolic syndrome, a condition that causes chronic complications and complex disease associations ranging from increased cardiovascular risk, chronic kidney disease, cancer and mental disorders, all reducing the quality of life [22,23,24]. Alterations in energy production are thought to be major contributors to the pathophysiology of these diseases and their complications [14,16,19]. 

The purpose of this manuscript is to provide a brief overview of the impact of metabolic diseases on mitochondrial respiration, as assessed by means of high-resolution respirometry, in both animal models and humans.

### High-Resolution Respirometry

Measuring mitochondrial respiration provides the most valuable insight into the cellular oxidative metabolism [25]. The gold standard in assessing the mitochondrial respiratory function was introduced more than half century ago and consists of the polarographic measurement of the oxygen consumption rates (OCR) in isolated mitochondria and cells with the aid of the Clark electrode [26]. High-resolution respirometry (HRR) provides nowadays the most accurate amperometric measurement of OCR by using closed air-tight reaction chambers and oxygen sensors with high sensitivity and increased precision [25,26,27]. With the introduction of the HRR method, the classic term “electron transport chain” has been replaced with more accurate terminology, namely the “electron transport system” (ETS) [27,28]. The efficiency of OXPHOS depends on the delivery of the reducing equivalents into the ETS as well as on the activities of the enzymatic complexes. The optimal efficiency and flow ratios are determined by control of complex I (CI) and II (CII). In HRR assays, the supply of CI and CII substrates (NADH and succinate-pathways) are most frequently used to achieve the convergent electron input at the Q-junction and the maximal OXPHOS and ETS capacities, respectively [29]. 

Specifically, samples are exposed to respiratory substrates, inhibitors and uncouplers in a standard order based on the ETS pathways, according to the so-called SUIT (Substrate-Uncoupler-Inhibitor Titration) protocols, which allows understanding of mitochondrial respiratory control in health and disease via the measurement of respiratory parameters [25,28]. Further analysis of mitochondrial respiratory dys/function is achieved by computing the flux control ratios (FCR) as ratios of oxygen flux in different respiratory control states, normalized for maximum flux corresponding to a reference respiratory state. FCR obtained from a SUIT protocol provide an internal normalization, expressing respiratory control independent of the mitochondrial content/markers for mitochondrial amount (e.g., mtDNA, citrate synthase, tissue protein or mass) and tend to be more comparable between different studies [29,30]. Since normalization is an important issue in the interpretation of the results in HRR experiments, the biomarker use for normalization is mentioned in the tables summarizing the literature data and is further examined in the Discussion section.

Current definitions of the respiratory parameters and several flux control ratios can be found in the publications of Gnaiger et al. at the bioblast/mitopedia site; those used in the current review are briefly summarized in Table 1.

**Figure 1:**
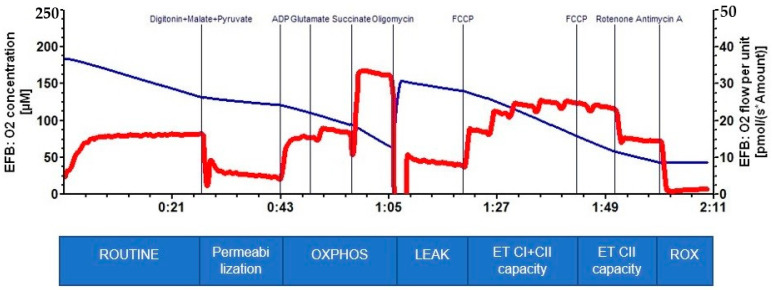
A representative trace of HRR in permeabilized cells (platelets) displaying the oxygen concentration in the chamber over time (blue trace) and the oxygen flux (red trace) according to the SUIT protocol. The additions are provided in the upper part of the chart. The respiratory parameters are provided in the lower part: ROUTINE respiration; OXPHOS capacity (the maximal active respiration in the presence of CI and CII substrates plus ADP); LEAK (the non-phosphorylating respiration in the presence of oligomycin); ET capacity (the maximal uncoupled respiration in the presence of FCCP); and ROX (residual oxygen consumption). See the explanations in the text above.

Figure 1 presents a typical HRR trace of permeabilized platelets recorded with the oxygraph O2-k (courtesy of Vlad Avram). In brief, platelets are added to the respiratory buffer inside the chambers, and cellular respiration is allowed to stabilize in order to measure the ROUTINE respiration (respiration in the physiological coupling state using endogenous substrates). The SUIT protocol starts after permeabilization of the plasma membrane with digitonin. In the presence of ADP and the conventional substrates for CI (pyruvate, glutamate, malate) and CII (succinate), the simultaneous, convergent electron flow into the Q-junction mimicks the action of the Krebs cycle in intact cells and allows the measurement of OXPHOS capacity (the maximum active respiration). The LEAK state (the non-phosphorylating respiration) is measured in the presence of oligomycin (the ATP synthase inhibitor). The ET capacity for CI and CII (the maximal uncoupled respiration as indicator of the maximal ETS activity) is further obtained by stepwise titration with the protonophore, FCCP (carbonylcyanide p-trifluoro-methoxyphenyl-hydrazone). Complex I activity is then inhibited by rotenone to assess the ET capacity for CII. Finally, complex III is inhibited with antimycin A, allowing the measure the residual oxygen consumption (ROX) due to processes other than OXPHOS (which are subtracted from the other respiratory rates) [31].

For a detailed description of the experimental protocols of HRR, readers are invited to review the excellent paper of Djafarzadeh and Jakob published in the Journal of Visualized Experiments [25].

## 2. Effects of Obesity on Mitochondrial Respiration in Animal Studies

### 2.1. Mitochondrial Respiration in the Striatal Muscle of Obese Animals

Hey-Mogensen et al. have investigated mitochondrial respiration in soleus muscle of Zucker rats by using HRR to determine the effects of age and obesity. Obesity did not alter mitochondrial OXPHOS in young animals, whereas it reduced the OXPHOS capacity in adult rats. Advanced age also had an independent effect on increasing mitochondrial H_2_O_2_ release in both lean and obese animals [32]. 

In contrast, Rodrigues et al. reported that basal mitochondrial respiration, LEAK respiration and non-mitochondrial oxygen consumption were unchanged in diaphragms of rats with overfeeding-induced obesity. Samples from the obese group presented higher OXPHOS and ET capacities, and the authors suggested the changes are the respiratory adaptation to an increased metabolic demand [33]. Overall, these data also imply that the persistent metabolic challenge becomes detrimental and leads to a bioenergetic decline in skeletal muscles of obese animals. 

### 2.2. Mitochondrial Respiration in the Liver of Obese Animals

Zhao et. al. used two models of obesity, a high-fat diet, and a combination of high-fat, high-fructose, high-cholesterol diets, in three male mice strains (SPF, B6 or D2). HRR was performed on liver tissue harvested at 4, 8, 12 and 18 weeks on these diets. The D2 mice fed with high-fat diet presented with lower ET capacity accompanied by a reduction in OXPHOS capacity from 8 weeks onward, suggesting that the liver oxidative metabolism in this strain is sensitive to excessive fat. In contrast, OXPHOS capacity was lower in obese B6 mice fed with high-fat diet only at the 4-week time point, after which respiratory rates were similar to those of the control livers. However, a high-fat, high-fructose, high-cholesterol diet had a strong impact on liver oxidative metabolism in the male B6 mice, which presented lower ET capacity, LEAK respiration and OXPHOS capacity. All three strains showed lower RCR values, suggesting that mitochondrial uncoupling was a common feature in liver mitochondria of the overfed mice. The RCR reduction occurred at different time points during the study and was correlated with the development of non-alcoholic steatohepatitis (NASH) features in liver, suggesting that mitochondrial uncoupling may be a pathomechanism of this condition [34]. 

### 2.3. Mitochondrial Respiration in the Heart of Obese Animals

Boardman et al. used a diet-induced obese mouse model and assessed mitochondrial respiration in the murine hearts using the HRR technique. These authors reported decreased NADH-linked ROUTINE respiration, impaired NADH-linked OXPHOS capacity and unchanged RCR [35]. Similar results were found by Guarini et al. in Zucker rat hearts demonstrating impaired OXPHOS and ET capacities with no change in LEAK respiration and RCR [36]. These data indicate that while oxidative metabolism is decreased in the hearts of obese rodents, the mitochondrial coupling capacity is maintained. 

Wang et al. measured mitochondrial respiratory capacities in the hearts of ob/ob vs. wild-type mice on either a regular chow or high-fat diet across four age groups to investigate the impact of diet and age on mitochondrial function. No difference was found in NADH-linked OXPHOS or ROUTINE respiration. When the 2- and 4-month-old groups were compared, there was a significant effect of high fat diet on increasing mitochondrial capacity to oxidize fatty acids in younger animals. In addition, the high-fat diet increased ROUTINE respiration in 2-month-old wild-type mice, but not in ob/ob mice. The same pattern was also observed in NADH-linked OXPHOS, suggesting a limitation of respiration at complex I in these rodents. Reduced RCR due to a high-fat diet suggests an increase in mitochondrial uncoupling [37]. Table 2 presents a summary of the main HRR studies in obese animals.

## 3. Effects of Diabetes on Mitochondrial Respiration in Animal Studies

### 3.1. Mitochondrial Respiration in the Skeletal Muscle of Diabetic Animals

Holmstrom et al. showed that NADH-linked OXPHOS, OXPHOS capacity, ET capacity and succinate-linked ET capacity are increased in the extensor digitorum longus muscles of diabetic (*db*/*db*) mice. In contrast, in soleus muscle, the NADH-linked OXPHOS was significantly decreased, whereas all the other parameters had a similar trend. These data suggest that mitochondrial metabolism in different types of muscle is differently affected by diabetes, an observation that was reported in both rodents and patients with diabetes [38,39,40].

Wessels et al. found no difference in the OXPHOS state supported by CI and CII substrates when comparing lean non-diabetic *fa*/+ and obese diabetic *fa*/*fa* adult male ZDF rats. The RCR for CII supported respiration was unchanged while that for CI supported respiration was increased in lean animals, suggesting uncoupling [41]. Fink et al. assessed mitochondrial function at different ADP concentrations and observed a rightward shift in the relationship between mitochondrial oxygen consumption and ADP concentration in diabetic rats. This observation indicates that diabetic mitochondria require more ADP to produce ATP, and when the optimal ADP concentration is not available, less ATP will be produced, supporting the concept that ADP concentration is a limiting factor for OXPHOS [42]. 

In a comprehensive study, Alimujiang et al. have investigated the mitochondrial function in both muscle and liver of animal models with different types and stages of diabetes. T1D was induced by streptozotocin (STZ) injection. The *db*/*db* mice were used as a T2D model, and high-fat diet-induced obesity represented a pre-diabetic stage of T2D. In early stages of T1D and T2D no changes was observed in NADH-linked OXPHOS, succinate-linked OXPHOS, NADH-linked ET capacity and succinate-linked ET capacity, suggesting that the decline in mitochondrial metabolism in the muscles depends on the duration of diabetic metabolic disturbances [43].

### 3.2. Mitochondrial Respiration in the Liver of Diabetic Animals

In the same elegant study, Alimujiang et al. also investigated mitochondrial function in livers harvested from mice with different types and stages of diabetes. At the early stage of T1D, liver mitochondrial respiration was increased as shown by elevated NADH-linked OXPHOS, succinate-linked OXPHOS, NADH-linked ET capacity and succinate-linked ET capacity, whereas in the late stages, respiration was only slightly increased or comparable with the control group. Similar observations were reported for T2D in both early and late stages, with the latter showing statistically significant increases for succinate-linked ET capacity [43]. 

Bouderba et al. conducted a study on Psammomys obesus, a model of nutritional diabetes; adult animals develop insulin resistance when fed a standard laboratory chow, which is considered hypercaloric compared with their natural food. In liver mitochondria isolated from the diabetic animals, ROUTINE respiration with CI substrates and NADH-linked OXPHOS were decreased, whereas the succinate pathway remained largely unaffected. Even though LEAK respiration was not modified by the diabetic state, uncoupling was shown by the decrease in RCR [44]. 

Yin et al. harvested liver samples from *db*/*db* mice and found that diabetic livers presented a significant decrease in mitochondrial oxygen consumption mediated by CI, CII and CIV [45].

Holmstrom et al. reported that LEAK respiration and OXPHOS capacity were unchanged, whereas ET capacity and succinate-linked ET capacity were lower in diabetic mice. The L/E coupling control ratio was comparable between the two groups, whereas the P/E control ratio was increased, indicating that the OXPHOS in liver tissue of diabetic mice is less limited by the phosphorylating system [28,38]. 

Franko et al. aimed to delineate the contribution of insulin resistance versus the long-term adaptation of mitochondrial respiration to the metabolic challenge. Mice were either fed with a high-fat diet to induce obesity and T2D, had a genetic defect in insulin signaling causing systemic insulin resistance but not full-blown diabetes (IR/IRS-1+/mice), or were treated with streptozotocin to induce T1D. The response of liver mitochondria to the three metabolic challenges was different. For example, the high-fat diet mice showed similar NADH-linked and succinate-linked respiration to the controls. The insulin resistant mice had no change in NADH-linked respiration, but decreased succinate-linked respiration. In contrast, the T1D model animals exhibited increased NADH-linked respiration and unmodified succinate-linked respiration [46].

In conclusion, in animal models, liver mitochondrial respiration varies not only with the type of diabetes but also with the duration of disease [43,46]. 

### 3.3. Mitochondrial Respiration in the Heart of Diabetic Animals

The bioenergetic impairment in diabetic hearts (in animal models and humans) has recently been investigated in light of the diabetic cardiomyopathy pathomechanisms [47]. Marciniak et al. compared drug- and diet-induced models of diabetes in terms of metabolic features and mitochondrial functions. Mice were fed with regular chow or a fat-enriched diet for 3 weeks, and then were randomized to receive either streptozotocin or citrate injections. Mice fed with the regular chow diet and injected with streptozotocin did not develop mitochondrial respiratory defects. In contrast, both groups of high-fat diet fed mice (injected or not with streptozotocin) showed a reduction of NADH-linked OXPHOS in the heart [48]. Furthermore, Gupte et al. conducted a study to determine the effects of diet and age on cardiac mitochondrial function. Adult insulin resistant mice fed a high-fat diet presented increased NADH-linked and succinate-linked OXPHOS. The addition of rotenone (CI inhibitor) led to a slightly higher respiration in high-fat diet fed insulin resistant mice, suggesting that the succinate oxidation is enhanced by the metabolic challenge [49]. However, since RCR was increased indicating uncoupling, mitochondrial respiration is inefficient and does not result in higher ATP generation [28,49]. In contrast, Parker et al. reported a decrease in succinate-linked OXPHOS in diabetic rat hearts [50]. Similarly, other authors have found that heart mitochondrial respiration is impaired in diabetic rats, presenting reduced NADH-linked OXPHOS, OXPHOS capacity and decreased ET capacity [51,52,53,54]. 

Mitochondrial respiration is considered a key factor in the development of complications in diabetes. Watala et al. used a rat model to decipher this pathogenic link [55,56,57]. These experiments yielded interesting results, namely that diabetic heart mitochondria presented a lower RCR and higher L/E control ratio, thus indicating that diabetes induces cardiac uncoupling. The fact that the diabetic group was associated with a lower ADP/O ratio further suggests that these mitochondria produce less ATP than healthy controls [57]. The calculated P/E control ratio in diabetic heart mitochondria was close to 1, indicating that OXPHOS equals the ET capacity and lack of respiratory reserve [28,57]. In contrast, in NOD mice, a model of T1D, Schleier et al. reported that heart mitochondria showed higher RCR and lower LEAK respiration with no change in maximal OXPHOS compared with their controls, which suggests that the diabetic mitochondria are better coupled in this experimental model [58].

Kiebish et al. found that transgenic cardiac myocyte-specific cardiolipin synthase (CLS) mice presented lower mitochondrial respiratory rates than those of wild type mice after developing STZ-induced T1D [52]. Collectively, all these data suggest that the impact of diabetes on mitochondrial respiratory function varies among species.

Jayakumari et al. investigated the cardiac mitochondrial substrate utilization in diabetic mice at 2 and 10 weeks of hyperglycemia, respectively. They found that the 2-week (but not the 10-week) diabetic mice presented a reduction in both CI and CII-dependent OXPHOS and also in OXPHOS supported by CII alone. At 10 weeks of diabetes, there was no difference in the OXPHOS capacity between the groups. These specific changes in mitochondrial respiration supported by different energetic substrates may reflect differences in the expression of OXPHOS complexes since the authors reported a decreased expression of both CI and CII at 2 weeks (but not at 10 weeks) of diabetes [59].

MacDonald et al. evaluated the effects of diabetes and age upon mitochondrial respiration of saponin-skinned fibers dissected from the subendocardium and subepicardium of the left ventricles of Wistar rats. Diabetic rats had lower OXPHOS capacity with an associated decrease in NADH-linked OXPHOS; the results were similar when normalizing to either weight of muscle tissue or the activity of citrate synthase (CS), a mitochondrial marker enzyme. In addition, both ET capacity and CII-dependent ET capacity were impaired by the diabetes status. In addition, mitochondrial function was further compromised by uncoupling as CI-dependent LEAK respiration was found increased, an observation that was confirmed by the decreased CI-dependent RCR, but CII-dependent LEAK respiration was decreased [54]. These authors concluded that age leads to a similar impairment in CI substrate phosphorylation, an observation which has been confirmed by other groups [51,52,54]. This similarity between diabetes and aging led to the hypothesis that mitochondrial dysfunction contributes to the increased prevalence of diabetes and its complications with age [60,61,62].

### 3.4. Mitochondrial Respiration in Other Tissues of Diabetic Animals—Insights into Chronic and Acute Complications

Long term diabetes is associated with several complications such as diabetic retinopathy (DR) or nephropathy (DN) [23,63].

Nowadays there is unequivocal evidence that the microvascular disease in DR is partly due to the impaired bioenergetics in the neural retina, as recently reviewed [64]. Santos et al. [65] reported a decade ago that in the early stages of T1D, increased mtDNA biogenesis and repair compensates for the ROS-induced damage, but, at 6 months of diabetes, this mechanism is overwhelmed, and damage of mtDNA and ETS occurs.

Han et al. investigated mitochondrial respiration in retinal homogenates from the Nile rat and found that upon normalizing to maximal OXPHOS, the capacity flux control ratio (FCR) for the NADH pathway was impaired by sustained hyperglycemia (18 months), whereas the succinate pathway presented increased FCR in the same setting. The coupling control ratio was increased at 6 months of hyperglycemia but reached the values of the control group at 18 months of hyperglycemia. However, these authors also reported that compensatory changes in OXPHOS can be detected in retina as early as 2 months, prior to the development of hyperglycemia, and they were associated with the impairment of the mitochondrial outer membrane integrity [66].

Regarding DN, there are consistent findings of ETS defects, and a dichotomic behavior has been reported in tubular (but not in the glomerular) mitochondria with the progression of the disease. Thus, OXPHOS was increased during the early phases of experimental diabetes in mitochondria isolated from the renal cortex and proximal tubular cells and declined with the progression of albuminuria; however, the oxygen consumption rates were decreased in mitochondria harvested from glomeruli and podocytes, regardless the diabetic stage. An early increase in OXPHOS has been reported in diabetic cardiomyocytes (not only in the renal tubules) and has been considered an adaptive change to the excess of energetic substrates [67].

Serralha et al. investigated DN-elicited changes in mitochondrial respiration in the kidneys of diabetic rats, and found there was a significant decrease in mitochondrial oxygen consumption and RCR in the presence of CI substrates (malate and pyruvate), whereas the respiratory rates were unchanged with succinate, the CII substrate [68]. Similarly, Christensen et al. found decreases in CI-supported mitochondrial OXPHOS and total OXPHOS without changes in RCR in the diabetic rat kidney. LEAK respiration was increased in the kidney homogenates harvested from diabetic animals [69]. 

Hypoglycemia is the most common acute complication associated with the treatment of diabetes and has a strong impact on brain metabolism and function [70,71,72]. Hippocampal homogenate was used as a source of brain mitochondria by He et al. who reported that recurrent hypoglycemia in diabetic rats caused a drop in NADH-linked OXPHOS, OXPHOS capacity, ET capacity and succinate-dependent ET capacity compared with diabetic controls without recurrent drops in glycemia. Even though the NADH-linked pathway dependent LEAK respiration also decreased, the ATP concentration was low due to recurrent hypoglycemias [73]. The main findings of the above-mentioned studies are summarized in Table 3.

## 4. Effects of Obesity on Mitochondrial Respiration in Human Studies

### 4.1. Mitochondrial Respiration in Skeletal Muscle of Obese Patients

Conflicting data are available in the literature regarding the bioenergetic impairment in human skeletal muscles. Thus, Vijgen et al. found that skeletal muscle mitochondrial respiration is decreased in morbidly obese patients. Specifically, OXPHOS capacity supported by both CI and CII substrates was significantly lower than in lean controls, while LEAK respiration was unchanged [74]. In a study carried out by Phielix et al., similar changes in mitochondrial respiratory function were found in the permeabilized muscle fibers of non-diabetic obese and diabetic obese patients compared with non-diabetic lean controls. Non-diabetic obese subjects presented impaired mitochondrial NADH-linked respiration, decreased total OXPHOS capacity along with diminished maximal noncoupled respiration, i.e., ET capacity [75]. In contrast, Ara et al. reported unchanged mitochondrial respiratory function of muscle fibers harvested from deltoid and vastus lateralis muscles of obese subjects vs. lean controls [76]. 

Weight loss intervention did not lead to significant differences in mitochondrial OXPHOS or LEAK respiration for morbidly obese patients in comparison with lean controls, although NADH-linked respiration significantly increased after weight loss. In addition, the contribution of LEAK respiration to OXPHOS was diminished by weight loss [74]. Coen et al. showed that exercise and weight loss not only improve insulin sensitivity, but also increase mitochondrial NADH-linked respiration, OXPHOS capacity and ET capacity, and Fiorenza et al. have shown improvements in OXPHOS coupling efficiency [77,78]. This further adds to the great body of evidence that suggests that weight loss and physical exercise are key factors in the treatment and prevention of T2D via improvements in mitochondrial energetic metabolism [79,80].

### 4.2. Mitochondrial Respiration in Liver of Obese Patients

Lund et al. compared the hepatic mitochondrial OXPHOS capacity in obese and non-obese human subjects by using perioperative liver biopsies. They reported that OXPHOS mediated by complex I, II and IV and the CS activity did not differ when comparing the two groups [81]. 

Koliaki et al. directly quantified mitochondrial respiration in liver biopsies of obese insulin-resistant humans without or with histologically proven non-alcoholic fatty liver (NAFL) or with non-alcoholic steato-hepatitis (NASH) and compared with that of lean individuals. Despite similar mitochondrial content, obese humans, disregarding the NAFL status, had 4.3- to 5.0-fold higher maximal respiration rates compared with lean subjects. However, based on the decreased RCR and increased L/E coupling control ratio, liver mitochondria from obese subjects were uncoupled. The ET capacity had a positive correlation with serum glucose and triglycerides and a negative correlation with insulin sensitivity [82]. 

In conclusion, these data suggest that obesity complicated with liver pathology such as NAFL or NASH is associated with increased liver oxidative metabolism that most likely does not translate into increased ATP generation due to uncoupling [82].

### 4.3. Mitochondrial Respiration in Adipose Tissue of Obese Patients

Kraunsoe et al. used HRR to quantify mitochondrial respiration in human abdominal subcutaneous and intra-abdominal visceral (omentum majus) adipose tissue from biopsies obtained from obese patients undergoing bariatric surgery. Visceral fat contained more mitochondria per milligram of tissue than subcutaneous fat, but the cells were smaller. NADH-linked respiration, OXPHOS capacity and ET capacity were higher in visceral adipose tissue than in subcutaneous fat when normalizing to tissue weight. However, when expressed per mtDNA, visceral adipose tissue had significantly lower mitochondrial respiration [83]. Substrate control ratios were higher and the coupling control ratio lower in visceral compared with subcutaneous adipose tissue, suggesting mitochondrial uncoupling [28,83]. Mitochondrial uncoupling has been causally linked to the onset of diabetes and visceral abdominal fat and is a predictor of insulin resistance, further supporting the hypothesis that mitochondrial dysfunction may contribute to the transition between obesity and diabetes mellitus [84,85].

### 4.4. Mitochondrial Respiration in Myometrum of Obese Patients

Gam et al. aimed to investigate whether pre-pregnancy obesity alters or can predict alterations in the mitochondrial phenotype in human myometrium at term. Oxygen consumption assessed as OXPHOS capacity, NADH-and rotenone-inhibited succinate supported OXPHOS was unchanged. However, the RCR was on average 20% lower in the obese group compared with the controls, suggesting the presence of mitochondrial uncoupling [86]. In Table 4, data regarding the main HRR findings in obese patients are summarized.

## 5. Effects of Prediabetes on Mitochondrial Respiration in Humans

Szczerbinski et al. evaluated mitochondrial respiration in skeletal muscle and adipose tissue of patients with prediabetes, defined as either fasting hyperglycemia or fasting hyperglycemia combined with an impaired glucose tolerance test. Mitochondrial respiration was assessed in muscle before and after exercise intervention. Prior to the intervention, prediabetic patients presented no difference in NADH-linked OXPHOS, maximum OXPHOS or ET capacity when compared with controls with normal blood glucose parameters. Increase in exercise yielded no differences in mitochondrial respiratory parameters in skeletal muscle. In contrast, in adipose tissue, physical exercise for 3 months increased NADH-linked OXPHOS, maximum OXPHOS and ET capacity. The fact that exercise significantly improved mitochondrial metabolism only in adipose tissue (and not in skeletal muscle) strongly suggests that its impact is tissue specific and not related to the prediabetic state [87]. In conclusion, prediabetes apparently does not impact on cellular respiration (Table 5).

## 6. Effects of Diabetes on Mitochondrial Respiration in Humans

### 6.1. Mitochondrial Respiration in Skeletal Muscle of Diabetic Patients

Mitochondrial respiration normalized per milligram of muscle weight was significantly lower in patients with T2D compared with controls [40,88,89,90,91]. Specifically, NADH-linked OXPHOS and OXPHOS capacity were significantly decreased, and ET capacity declined [40,75,88,89,90,92,93]. However, Lund et al. reported no differences in mitochondrial respiratory rates in skeletal muscle of diabetic obese versus non-diabetic obese patients when normalized to both tissue weight and citrate synthase activity [94]. This was confirmed by another study in which the OXPHOS and ET capacities were normalized per citrate synthase activity, as a measure of mitochondrial content [40,88,89]. In contrast, Phielix et al. reported that OXPHOS capacity was reduced in diabetic patients, even when normalized to mtDNA copy numbers as an indicator of mitochondrial content [90].

Rabol et al. compared mitochondrial respiration and markers of mitochondrial content in the skeletal muscle of the arm and leg in patients with diabetes mellitus and obese control subjects. Mitochondrial respiration was measured by HRR from biopsies of deltoideus and vastus lateralis and showed variations between the two muscles. The differences in OXPHOS in the leg muscles were seen with CI-dependent substrates and CI^+^II-dependent substrates. The substrate control ratio for succinate was calculated and the ratio between state 3 with malate + glutamate + pyruvate + succinate and state 3 with malate + glutamate + pyruvate was compared to determine whether CI was responsible for the decrease in mitochondrial respiration. It was found that variations in respiration cannot be solely explained by modifications in NADH-linked respiration. These authors did not observe differences in mitochondrial respiration in the arm muscle between the groups; however, they found that respiration was lower in arm muscle compared with leg muscle [40]. Comparable results were reported by Larsen et al., i.e., state 3 CI-dependent mitochondrial respiration normalized to muscle tissue weight was decreased significantly in vastus lateralis of diabetic patients compared with obese non-diabetic controls, whereas that in deltoideus muscle was not changed [39]. When mitochondrial respiration was normalized to citrate synthase activity (as a marker of mitochondrial content), no differences were found between diabetic and control groups. However the calculated mitochondrial respiration “per individual mitochondria” with electron flux through both NADH and the succinate pathways was significantly higher in the arm muscles than leg muscles in the control group [40]. Larsen et al. attributes the reduction in mitochondrial respiration in leg muscles in diabetic patients mainly to the decreased mitochondrial content as indicated by a lower CS activity compared with controls [39]. These findings are in line with the concept that mitochondrial respiration and metabolic needs must match the energy demand, which is different between upper and lower body musculature, a difference that may be a consequence of evolution due to walking upright [39,40,95]. 

Antoun et al. examined oxidative phosphorylation and the ETS supercomplexes assembly in rectus abdominis muscles of obese diabetic vs. obese non-diabetic individuals. Mitochondrial respiration in permeabilized rectus abdominis muscle fiber was assessed using HRR. NADH-linked respiration, OXPHOS capacity and ET capacity were decreased in muscle samples from diabetic patients. No differences were observed in LEAK respiration (non-phosphorylating mitochondrial respiration) in the absence of ADP or in the presence of oligomycin (ATP synthase inhibitor). Notably, with the exception of LEAK respiration, there was an inverse correlation between HbA1c and mitochondrial respiratory rates in both diabetic and non-diabetic human subjects [96]. A similar inverse correlation between HbA1c and OXPHOS capacity was shown earlier by Mogensen et al. [91]. 

Exercise training is a key therapeutic strategy in diabetes that functions by increasing insulin sensitivity and providing glycemic control. Phielix et al. reported that physical exercise also improved the NADH-linked OXPHOS, OXPHOS capacity and ET capacity in skeletal muscle of non-diabetic and diabetic patients with the same BMI. Exercise training (12 weeks) increased NADH-linked respiration by 34%, OXPHOS capacity by 33% and ET capacity by 33% in the diabetic patients. However, an increase in LEAK respiration (28%) was also observed, suggesting that the increased respiration does not necessarily lead to an efficient translation into more ATP generation. In addition, upon normalization to mtDNA copy number, the increase in respiration due to training disappeared, indicating that the improvement of mitochondrial metabolism in muscle fibers is attributed to an increase in total number of mitochondria due to physical exercise [79,80,93].

### 6.2. Mitochondrial Respiration in Liver of Diabetic Patients

The data reported by Lund et al. on the OXPHOS capacity of human liver samples do not support differences in the CI, CII and CIV-linked respiration in either obesity or diabetes compared with controls; in addition, citrate synthase activity as an assessment of mitochondrial content also was unchanged [81]. 

### 6.3. Mitochondrial Respiration in Adipose Tissue of Diabetic Patients

Hansen et al. aimed to study adipose tissue mitochondrial respiration and lipolysis in patients with diabetes and obesity compared with non-diabetic controls following extensive weight loss by diet and a Roux-en-Y gastric bypass. The mitochondrial respiratory rates were similar. With normalization to the mitochondrial content, no differences in oxidative capacity after gastric bypass were seen. The P/E control ratio increased 18 months after surgery in both groups, indicating that the OXPHOS capacity becomes less limited by the phosphorylation system with weight loss [28,97]. Bódis et al. harvested adipose tissue (both superficial and deep) and assessed mitochondrial respiration by HRR. Although respiratory rates were similar between diabetic patients and healthy controls, a degree of mitochondrial uncoupling was found in the diabetic group in both adipose tissue types, as indicated by the decrease in RCR and increase in the LEAK control ratio [98]. These observations are in line with the fact that uncoupling was reported solely in visceral adipose tissue of obese as well as diabetic patients, and the percentage of visceral fat mass relative to the total body fat was higher compared with that of the obese non-diabetic group [83,98]. Uncoupled respiration is inefficient as it dissipates the protonomotive force; subsequently, the coupling efficiency of OXPHOS is lower which, in turn, leads to decreased insulin secretion and/or activity [14,28,99].

### 6.4. Mitochondrial Respiration in Hearts of Diabetic Patients

Montaigne et al. studied the effects of obesity, insulin resistance and diabetes on contractility and mitochondrial function of the human myocardium before the onset of cardiomyopathy. NADH-linked OXPHOS in atrial myocardium was decreased in diabetic patients without signs of cardiomyopathy, whereas succinate-linked OXPHOS was decreased only in diabetic patients with normal weight (no change for overweight or obese diabetic patients) [100]. Diabetes was associated with a poorly coupled respiration, as demonstrated by the low RCR. These changes in mitochondrial respiration are similar to thosse reported in skeletal muscle [90,93,96]. In contrast, the obese status was not associated with altered cardiac mitochondrial respiratory rates, unlike in skeletal muscle [74,75,100]. 

Duicu et al. assessed mitochondrial oxygen consumption of atrial fibers harvested from patients with coronary heart disease and diabetes. The group showed that mitochondrial respiration was inhibited both through the NADH-linked and succinate-linked pathway, leading to an all-round decrease in mitochondrial respiratory rates compared with controls without coronary heart disease. NADH-linked respiration was more severely affected in the diabetic group [101], which potentially may expose these patients to increased oxidative stress [66,102]. Oxidative stress in the diabetic human hearts may be raised by the increased expression of monoamine oxidase (in particular, the MAO-B isoform) that has been associated with mitochondrial dysfunction [103] and a significant source of oxidative stress in diabetes [104].

### 6.5. Mitochondrial Respiration in Platelets of Diabetic Patients

Circulating platelets offer a promising primary tissue alternative to biopsies for the study of mitochondrial bioenergetics in both acute and chronic diseases [105]. Mitochondrial respiration in permeabilized human platelets was studied by Gvozdjakova et al. in patients with chronic kidney disease and various comorbitidies, including a subgroup with diabetes, and no significant differences were found compared with healthy volunteers [106]. In a pilot study, we also reported no differences in respiratory rates of both intact and permeabilized platelets between diabetic patients and healthy controls; specifically, there were no differences in LEAK respiration, E-L coupling efficiency, R-L control efficiency (performed only in intact platelets) or P-L control efficiency (performed only in permeabilized platelets), suggesting that there is no difference in the degree of uncoupling between the two groups [107]. Whether diabetes has a lower impact on platelet mitochondrial respiration compared with that in muscle or adipose tissue remains to be confirmed by large, multicentric studies. Table 6 summarizes the main HRR changes in diabetic patients.

## 7. Discussion

### 7.1. General Discussion

The results of studies on mitochondrial bioenergetics in overfeeding-induced obesity and diabetes are often contradictory in different tissues and in animal models. It has been hypothesized that the type of diet and diet components introduce confounding variables. Gonzalez-Armenta et al. aimed to determine the effects of dietary patterns on oxidative metabolism in cynomolgus macaques. NADH-linked OXPHOS, OXPHOS capacity, ET capacity and succinate-linked ET capacity were higher upon overfeeding. In addition, mitochondrial respiration in response to fatty acids was significantly and positively correlated with both insulin resistance and hyperinsulinemia, an observation that has been previously confirmed by other authors [18,108,109]. Similar modifications in mitochondrial respiration have been reported using high-fat diet models [37,46,49]. Baldini et al. used 3T3-L1 mouse fibroblasts matured into adipocytes and challenged with long-chain fatty acids as a model to mimic obesity in in vitro settings. HRR studies revealed that hypertrophic adipocytes had a comparable ROUTINE respiration to the normal mature adipocytes, while presenting decreased NADH-linked OXPHOS, OXPHOS-capacity and ET capacity, as reported in an earlier study of Zhao et al. [34,110]. A possible explanation may be that a diet rich in fatty acids causes an increased mitochondrial-dependent oxidative stress, leading to mitochondrial damage that, in turn, activates the mitochondrial quality control mechanisms, mitophagy, with subsequent reduction in the mitochondrial content [14,40,89,111,112,113].

Hyperglycemia also has a different impact on mitochondrial respiration in skeletal muscle versus liver, with the latter being less and/or later affected. This may be explained by differences in metabolic substrates used by these two organs in various pathophysiological conditions and the specific glucose transporters, insulin-insensitive GLUT2 for liver and insulin-sensitive GLUT4 for muscle [43].

It is also important to note that CI^−^ and CI^+^CII-supported respiratory rates as well as ET capacity are inversely correlated with HbA1c, suggesting that poor glycemic control impairs mitochondrial function [91,96]. In addition, insulin secretion is also dependent on proper pancreatic mitochondrial function, while poor glycemic control due to decreased insulin amount and/or activity impairs systemic and local bioenergetics of other tissues, indicating a mutual relationship and two-way feedback mechanism between insulin and oxidative metabolism [14,91,96,99]. This hypothesis is supported by the fact that chronic hyperglycemia causes a phenomenon known as glucotoxicity, which causes defects in pancreatic β-cells due to mitochondrial stress and formation of reactive oxygen species [114,115] and advanced glycation end products, which reduce the mitochondrial coupling efficiency [114,116]. 

Phielix et al. reported a positive correlation between the suppression of lipid oxidation upon insulin stimulation and the respiratory quotient (RQ). Specifically, the RQ corelated with NADH-linked respiration, while the suppression of insulin-linked lipid oxidation corelated with NADH-linked OXPHOS, maximal OXPHOS and ET capacity. The same study shows positive correlations between maximal succinate stimulated H_2_O_2_ production and HbA1c levels. These results suggest that it is the mitochondrial oxidative metabolism rather than hyperglycemia that predicts the insulin-dependent change in the RQ. The association of metabolic flexibility with mitochondrial function and its lack of association with insulin sensitivity indicate that mitochondria are responsible for the diminished insulin-stimulated increase in the oxidation of substrates in subjects with insulin-resistance [75]. 

As suggested by Collins et. al., ROS are considered a double-edged sword for β-cell function [117]. In this regard, it is hypothesized that hyperglycemia will increase ROS production, leading to mitochondrial uncoupling via activation of uncoupling proteins in the pancreas, lower ATP production and decreased insulin synthesis, all resulting in a vicious circle that perpetuates hyperglycemia [14,117]. Oxidative stress contributes to the impaired insulin production via complex signal transduction that includes AMPK activation with subsequent harmful effects mediated by ERK activation and mTOR inhibition, both resulting in decreased β-cell proliferation [21]. Excessive ROS generation is responsible for both β-cell dysfunction and the development of insulin resistance [118], and a plethora of preclinical studies unequivocally demonstrated their role in the pathogenesis of diabetes and its complications, as recently reviewed in refs. [119,120]. However, most clinical trials (with few exceptions) failed to demonstrate long-term benefits of the antioxidant therapies [120], albeit in the short-term, several phytochemicals showed beneficial effects [121]. Importantly, it was reported one decade ago that mitochondrial ROS are critical for insulin secretion [122,123]; therefore, targeting their reduction had to be carefully managed. Moreover, certain drugs commonly used to treat diabetes, such as metformin and those used to treat associated conditions such as dyslipidemia (statins) or hypertension (valsartan, amlodipine) have been known to mitigate ROS production [124].

The effects of antidiabetic drugs on mitochondrial bioenergetics was not the topic of the present review. However, we would like to mention several drug-induced effects on mitochondrial respiration. Incubation of skeletal muscle with metformin, the cornerstone of T2D therapy, decreased the NADH-linked OXPHOS capacity, LEAK respiration and ET capacity in a dose-dependent manner [41]. Similarly, NADH-linked respiratory dysfunction induced by metformin in human platelet mitochondria has been shown by Piel et al. [125,126,127]. These observations indicate that at least some of the mitochondrial respiratory changes found in diabetic patients are not caused by the disease itself but also by the treatment. Other studies demonstrated that inhibition of mitochondrial CI with metformin and other compounds such as rotenone or berberine, led to lower glucose levels, suggesting that inhibition of CI may be somehow beneficial in the setting of diabetes [43,127,128,129,130,131]. However, a severely depressed NADH-linked respiration may lead to depressed OXPHOS and ATP generation, energy starvation and collapse of the ATP-dependent pancreatic insulin secretion [14,132]. It must be noted that pancreatic β-cells lack the levels of lactate dehydrogenase found in other cells such as muscle cells or pancreatic α-cells and as such do not operate glycolytically to generate ATP, which means that CI inhibition may lead to decreases in ATP-linked insulin secretion, potentially explaining the decrease in insulin secretion found in patients with long-term diabetes [132].

A potential solution would be to increase mitochondrial respiration via the succinate pathway to compensate CI inhibition, which could be achieved by the use of a novel class of prodrugs, the cell permeable succinates [125,133,134,135]. Last but not least, a novel mitochondriotropic drug in the treatment of diabetes that provides both CI inhibition and increases in succinate-linked respiration is imeglimin. It has the added benefit of reduced mitochondrial ROS production and increased mitochondrial content, both outcomes that may also delay the onset of chronic diabetic complications [136,137].

### 7.2. Normalization in HRR Studies

While variations in respiratory parameters are obviously dependent upon among species and tissues (Table 2, Table 3, Table 4, Table 5 and Table 6), and in some cases even within the same type of tissue in a pathological setting (as reported by Rabol et al. and Larsen et al. [39,40]), it has to emphasized that normalization when reporting (and comparing) the results should be taken into acount when performing HRR experiments. This can be achieved by various means, such as reporting respiratory rates relative to tissue protein or mass, number of cells or markers such as citrate synthase or mtDNA [28].

One relevant example in this respect when reporting data regarding mitochondrial respiration can be seen in the work of Boushel et al. where both OXPHOS and ET capacity in vastus lateralis samples harvested from diabetic patients were decreased when normalized to tissue weight, whereas upon normalizing to mtDNA content, the differences dissapeared [89].

Lack of uniformity in normalization is an important limitation when comparing studies using HRR; however, this can be overcome to a certain extent by calculating the flux control ratios [28].

### 7.3. Strengths and Limitations of HRR Technique

HRR offers higher sensitivity, has lower oxygen leak and requires smaller biological sample sizes while providing the opportunity for simultaneous comparative measurements in two chambers [25]. 

However, there are certain limitations such as [25,28]:

- The concentrations of substrates are different in living cells than in the experimental cocktails used in the experimental setting;
- Permeabilized cells or tissues and isolated mitochondria lack cytoplasm and the machinery to undergo glycolysis and other central metabolic pathways;
- Cell cultures differ from living cells as environmental factors alter mitochondial density (e.g., physical activity in skeletal muscles), making normalization a critical approach;
- Longer experiments may lead to oxygen depletion in the chamber, altering results (which can be somewhat solved by reoxygenating the chamber mid-experiment);
- Since the chambers are reused and experiments may vary, there is a risk of contamination with inhibitors. For this reason washing and cleaning procedures between experiments are essential, even if time consuming.

## 8. Concluding Remarks 

Accurate assessment of the mitochondrial respiration and ETS function by means of high-resolution respirometry has emerged as powerful tool for studying the pathophysiology of a myriad of diseases which impair cellular bioenergetics. The mechanistic understanding of the pathogenesis of mitochondrial-driven pathologies is a prerequisite for the discovery and use of novel biomarkers, such as bioenergetics of circulating blood cells, to inform clinical diagnosis and monitor treatment response in the era of personalized medicine.

## Figures and Tables

**Table 1:** Respirometry parameters and some control ratios assessed by HRR [28].

Parameter	Definition
ROUTINE respiration	Mitochondrial respiration at non-saturating levels of ADP
OXPHOS capacity	Respiratory capacity of mitochondria in an ADP-stimulated state at saturating levels of ADP
ET capacity	Mitochondrial respiration in the non-coupled state, achieved by titrating an optimum concentration of uncouplers (protonophores)
LEAK respiration	Non-phosphorylating state when oxygen flux is minimized by the backpressure of a high protonmotive force generated by ATP synthase inhibition
RCR	Respiratory control ratio. Calculated as OXPHOS capacity/LEAK respiration
UCR	Uncoupling control ratio. Calculated as ET capacity/Routine respiration
P/E control ratio	Phosphorylation system control ratio. Calculated as OXPHOS capacity/ET capacity
L/E coupling-control ratio	Flux ratio. Calculated as LEAK respiration/ET capacity

**Table 2:** High-resolution respirometry findings in animal obesity studies.

Animal Model	Tissue Type	Main Effects	Normalization	Ref.
Zucker rats	Skeletal muscle (Soleus muscle)	Decreased OXPHOS capacity in adult No change in OXPHOS capacity in young	Citrate synthase	[32]
Wistar rats	Skeletal muscle (left diaphragm)	No change in ROUTINE respiration No change in LEAK respiration No change in ROX Increased OXPHOS capacity Increased ET capacity	Tissue mass	[33]
SPF male B6 and D2 mice	Liver tissue	Decreased OXPHOS capacity Decreased ET capacity Decreased RCR	Tissue mass	[34]
C57BL/6J male mice	Heart muscle (Langendorff-perfused hearts)	Decreased ROUTINE respiration Decreased NADH-linked OXPHOS capacity No change in RCR	Tissue protein	[35]
Zucker rats	Heart muscle (Langendorff-perfused hearts)	Decreased OXPHOS capacity Decreased ET capacity No change in LEAK respiration No change in RCR	Tissue mass	[36]

**Table 3:** High-resolution respirometry findings in animal diabetes studies.

Animal Model	Tissue Type	Main Effects	Normalization	Ref.
C57BL/6J mice	Skeletal muscle (hindlimb muscle)	Peak respiration by the diabetic mitochondria required a higher level of ADP (right shift in the curve of O2 flux vs. ADP)	Tissue mass	[42]
db/db mice bred on a C57BL/6J background	Skeletal muscle (soleus; extensor digitorum longus)	*In soleus muscle:* Increased NADH-linked OXPHOS Increased OXPHOS capacity Increased ET capacity Increased succinate-linked ET capacity No change in LEAK respiration No change in L/E coupling control ratio No change in P/E control ratio *In extensor digitorum longus muscle:* Decreased NADH-linked OXPHOS No change in OXPHOS capacity No change in ET capacity No change in succinate-linked ET capacity Increased LEAK respiration No change in L/E coupling control ratio No change in P/E control ratio	Tissue mass	[38]
C57BL/6J mice (type 1 diabetes and control) C57BL/Ks db/db mice (type 2 diabetes)	Skeletal muscle (quadriceps femoris; biceps femoris; soleus; gastrocnemius)	*In early-disease type 1 or type 2 diabetes:* No change in NADH-linked OXPHOS No change in succinate-linked OXPHOS No change in NADH-linked ET capacity No change in succinate-linked ET capacity	Tissue mass	[43]
ZDF rats non-diabetic fa/+ ZDF rats obese, diabetic, fa/fa	Skeletal muscle (tibialis anterior)	No difference in NADH-linked OXPHOS No difference in CII supported respiration Increased RCR dependent on CI substrates No difference in RCR dependent on CII substrates	Tissue mass Control OXPHOS	[41]
db/db mice bred on a C57BL/6J background	Liver tissue	No change NADH-linked OXPHOS No change in OXPHOS capacity Decreased in ET capacity Decreased succinate-linked ET capacity No change LEAK respiration No change in L/E coupling control ratio Increased in P/E control ratio	Tissue mass	[38]
C57BL/6J mice (type 1 diabetes and control) C57BL/Ks db/db mice (type 2 diabetes)	Liver tissue	*At the early stage of type 1 diabetes:* Increased NADH-linked OXPHOS Increased succinate-linked OXPHOS Increased NADH-linked ET capacity Increased succinate-linked ET capacity *At the late stage of type 1 diabetes:* No change in NADH-linked OXPHOS No change in succinate-linked OXPHOS No change in NADH-linked ET capacity No change in succinate-linked ET capacity *At the early stage of type 2 diabetes:* Increased NADH-linked OXPHOS Increased succinate-linked OXPHOS Increased NADH-linked ET capacity Increased succinate-linked ET capacity *At the late stage of type 2 diabetes:* No change in NADH-linked OXPHOS No change in succinate-linked OXPHOS No change in NADH-linked ET capacity Increased succinate-linked ET capacity	Tissue mass	[43]
IR/IRS-1^+/−^ mice generated by crossing mice heterozygous for insulin receptor null and IRS-1 null alleles, respectively, into the C57BL/6N background	Liver tissue	*In high fat diet type 2 diabetic mouse model:* No change in NADH-linked ROUTINE respiration No change in succinate-linked ROUTINE respiration *In insulin-resistant mouse model (without diabetes):* No change in NADH-linked ROUTINE respiration Decreased succinate-linked ROUTINE respiration *In type 1 diabetic mouse model:* Increased in NADH-linked ROUTINE respiration No change in succinate-linked ROUTINE respiration	Tissue protein	[46]
Psammomys obesus	Liver tissue	Decreased NADH-linked ROUTINE respiration Decreased NADH-linked OXPHOS No change in succinate-linked ROUTINE respiration No change in succinate-linked OXPHOS No change in LEAK respiration Decreased RCR	Tissue protein	[44]
C57BLKS/J db/db and db/m mice	Liver tissue	Decreased NADH-linked OXPHOS Decreased succinate-linked OXPHOS Decreased CIV-dependent OXPHOS	Mitochondrial mass	[45]
FVB/N mice	Heart muscle (left ventricle)	No change in NADH-linked OXPHOS Decreased succinate-linked OXPHOS No change in LEAK respiration	Tissue mass	[50]
Ldlr^−/−^ and C57BL/6J mice	Heart muscle	Increased NADH-linked OXPHOS Increased succinate-linked OXPHOS	To controls	[49]
C57/BL6J mice	Heart muscle	Decreased NADH-linked OXPHOS	Tissue mass mtDNA copy number	[48]
Wistar rats	Heart muscle	Decreased RCR Higher LEAK control ratio Increased P/E control ratio	No information	[57]
Wistar rats	Heart muscle	Decreased NADH-linked OXPHOS Decreased OXPHOS capacity Decreased ET capacity Decreased RCR dependent on CI substrates No difference in RCR dependent on CII substrates	Tissue mass Citrate synthase	[54]
Wistar rats	Heart muscle	Decreased NADH-linked OXPHOS Decreased OXPHOS capacity Decreased ET capacity	Volume	[51]
C57BL/6 mice	Heart muscle	*At 2 weeks of diabetes:* Decreased NADH-linked OXPHOS Decreased succinate-linked OXPHOS Decreased OXPHOS capacity *At 10 weeks of diabetes:* No changes NADH-linked OXPHOS mediated by glutamate Increased NADH-linked OXPHOS mediated by glutamate and pyruvate No changes succinate-linked OXPHOS No changes OXPHOS capacity	Mitochondrial protein content	[59]
NOD/ShiLtJ mice and NOR/Lt mice	Heart muscle	No changes OXPHOS capacity Decreased LEAK respiration Increased RCR	Tissue mass	[58]
db/+ and db/db C57BL/Ks mice	Heart muscle	Decreased NADH-linked OXPHOS Decreased ET capacity No change in LEAK respiration	Tissue protein	[53]
wild type C57BL/6J mice and CLS mice	Heart muscle	Decreased NADH-linked OXPHOS Decreased OXPHOS capacity No change in LEAK respiration No change NADH-linked OXPHOS No change OXPHOS capacity No change in LEAK respiration	Tissue protein	[52]
Nile rats	Retina homogenate	Decreased FCR for NADH pathway Increased FCR for succinate pathway FCR normalized to OXPHOS capacity	Tissue mass	[66]
TRPC6 global knockout mice and wild type mice	Hippocampal neuron homogenate	Decreased NADH-linked OXPHOS Decreased OXPHOS capacity Decreased ET capacity Decreased succinate-dependent ET capacity Decreased NADH-dependent LEAK respiration	Tissue mass	[73]
Wistar rats	Kidney homogenate	Decreased ROUTINE respiration in the presence of CI substrates No change in ROUTINE respiration in the presence of CII substrate No change in LEAK respiration Decreased RCR dependent on CI substrates No difference in RCR dependent on CII substrates	Tissue mass	[68]
Sprague-Dawley rats	Kidney homogenate	Decreased NADH-linked OXPHOS Decreased OXPHOS capacity No change in RCR Increased LEAK respiration	Mitochondrial protein content	[69]

**Table 4:** High-resolution respirometry findings in human obesity studies.

Cell Type (Harvest Site)	Main Effects	Normalization	Ref.
Skeletal muscle (vastus lateralis)	Reduced NADH-linked OXPHOS Reduced OXPHOS capacity Reduced ET-capacity	Tissue mass	[75]
Skeletal muscle (deltoideus; vastus lateralis)	No change in NADH-linked OXPHOS No change in OXPHOS capacity No changes in ROX	Tissue mass	[76]
Skeletal muscle (quadriceps femoris)	Decreased OXPHOS capacity No change in ET capacity	Tissue mass	[74]
Liver tissue (lower part of right liver lobe)	Increased NADH-linked OXPHOS Increased OXPHOS capacity Increased ET-capacity Decreased RCR Increased L/E coupling control ratio	Tissue protein	[82]
Liver tissue (lower part of right liver lobe)	No change in NADH-linked OXPHOS No change in succinate linked OXPHOS No change in ET-capacity	Tissue mass	[81]
Adipose tissue (abdominal subcutaneous; intra-abdominal visceral from omentum majus)	No change in RCR Decreased UCR Visceral fat vs. subcutaneous fat	Number of cells mtDNA content	[83]
Myometrial biopsies	No change in NADH-linked OXPHOS No change in OXPHOS capacity Decreased RCR	No information	[86]

**Table 5:** High-resolution respirometry findings in prediabetes.

Cell Type (Harvest Site)	Main Effects	Normalization	Ref.
Skeletal muscle (vastus lateralis)	No changes in NADH-linked OXPHOS No changes in OXPHOS capacity No changes in ET capacity	Tissue mass Citrate synthase	[87]
Adipose tissue (subcutaneous; periumbilical area)	No changes in NADH-linked OXPHOS No changes in OXPHOS capacity No changes in ET capacity	Tissue mass Citrate synthase	[87]

**Table 6:** High-resolution respirometry findings in human diabetes studies.

Cell Type (Harvest Site)	Main Effects	Normalization	Ref.
Skeletal muscle (vastus lateralis)	Decreased OXPHOS capacity	Citrate synthase	[91]
Skeletal muscle (vastus lateralis)	Decreased NADH-linked OXPHOS Decreased OXPHOS capacity Decreased ET capacity	Tissue mass Citrate synthase	[88]
Skeletal muscle (vastus lateralis)	*When normalizing to tissue weight:* Decreased NADH-linked OXPHOS Decreased OXPHOS capacity Decreased ET capacity *When normalizing to mtDNA content:* No changes in NADH-linked OXPHOS No changes in OXPHOS capacity No changes in ET capacity	Tissue mass mtDNA content	[89]
Skeletal muscle (vastus lateralis)	Decreased OXPHOS capacity Decreased ET capacity	Citrate synthase mtDNA content	[90]
Skeletal muscle (deltoideus; vastus lateralis)	*In vastus lateralis:* Decreased NADH-linked OXPHOS Decreased OXPHOS capacity *In deltoideus:* No change in NADH-linked OXPHOS No change in OXPHOS capacity	Citrate synthase	[40]
Skeletal muscle (rectus abdominis)	Decreased NADH-linked OXPHOS Decreased OXPHOS capacity Decreased ET capacity	Tissue mass	[96]
Skeletal muscle (vastus lateralis)	Decreased NADH-linked OXPHOS Decreased OXPHOS capacity Decreased ET capacity	Tissue mass	[75]
Skeletal muscle (deltoideus; vastus lateralis)	*In deltoideus muscle:* No changes NADH-linked OXPHOS No changes in OXPHOS capacity *In vastus lateralis muscle:* Decreased NADH-linked OXPHOS Decreased in OXPHOS capacity	Citrate synthase	[39]
Skeletal muscle (vastus lateralis)	Decreased NADH-linked OXPHOS Decreased OXPHOS capacity	Tissue mass Citrate synthase	[92]
Skeletal muscle (vastus lateralis)	Decreased NADH-linked OXPHOS Decreased OXPHOS capacity Decreased ET capacity	mtDNA content	[93]
Skeletal muscle (vastus lateralis)	No change in OXPHOS capacity No change in ET capacity	Citrate synthase	[94]
Adipose tissue (subcutaneous paraumbilical region: superficial—above the fascia Scarpa; deep—beneath the fascia Scarpa)	No change in NADH-linked OXPHOS No change in OXPHOS capacity No change in ET capacity Decreased RCR Decreased LEAK control ratio	Citrate synthase	[98]
Adipose tissue (subcutaneous abdominal region)	No change in RCR	Tissue mass mtDNA content	[97]
Heart muscle (right atrium)	Decreased NADH-linked OXPHOS Decreased succinate-linked OXPHOS Decreased RCR	Citrate synthase	[100]
Heart muscle (right atrium)	Decreased NADH-linked OXPHOS Decreased NADH-linked ET capacity Decreased NADH-linked LEAK respiration Decreased succinate-linked OXPHOS Decreased succinate-linked ET capacity Decreased succinate-linked LEAK respiration	Volume Citrate synthase	[101]
Platelets (venous blood)	No change in NADH- and succinate linked OXPHOS No change in ET capacity No change in succinate-dependent ET capacity	Number of cells	[106]
Platelets (venous blood)	No change in NADH-linked OXPHOS No change in OXPHOS capacity No change in succinate-linked ET capacity No change in ET capacity No change in LEAK respiration No change in R-L control efficiency (intact) No change in P-L control efficiency (permeabilized) No change in E-L coupling efficiency	Number of cells	[107]

## Data Availability

Not applicable.
